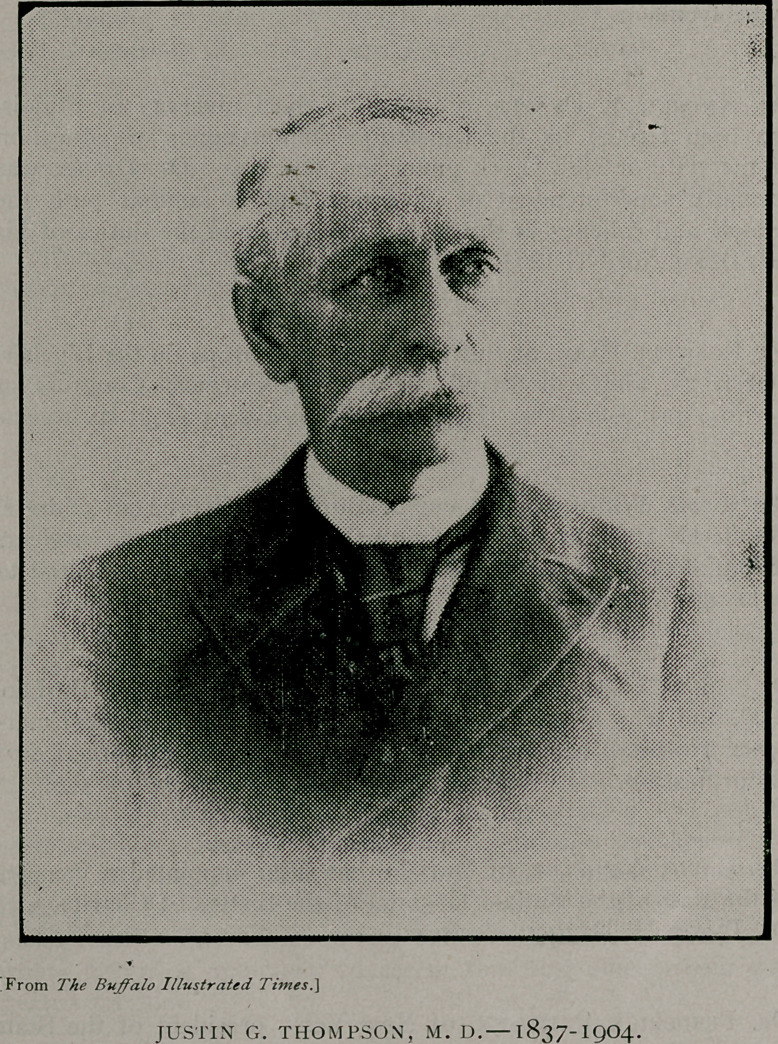# Dr. Justin G. Thompson

**Published:** 1904-04

**Authors:** 


### OBITUARY.

Dr. Justin G. Thompson, of Angola, N. Y., died at his home in that village March 6, 1904, aged 66 years. He had been long ill

[From *The Buffalo Illustrated Times.*]

JUSTIN G. THOMPSON, M. D.—1837-1904.

with heart complications consecutive to recurrent attacks of grip and finally died from heart failure.

Dr. Thompson was a native of Chautauqua County, received his preliminary education at Fredonia Academy and graduated in medicine at Ann Arbor, Mich., in 1861. Soon afterward, September 5, 1861, he was commissioned second lieutenant of Company G, 49th N. Y. Infantry. Later in the autumn he suffered an attack of typhoid fever which caused his resignation December 26, 1861. He was commissioned assistant surgeon of the 9th N. Y. Cavalry, August 11, 1862; was appointed assistant surgeon 77th N. Y. Infantry, November 17, 1862, was promoted surgeon of the same regiment December 17th, 1864, and was mustered out with the regiment June 27, 1865. During the campaign in the wilderness he was left in charge of wounded and became a prisoner, remaining on that duty about forty days, after which he returned to his command.

Soon after the war ended Dr. Thompson located at Angola, where he engaged in active professional practice which continued until his last sickness. He took active interest in the affairs of the Grand Army of the Republic and for several years was commander of the post where he lived; also, he was master of the masonic lodge at Angola for a number of years. He was surgeon of the Lake Shore and Michigan Southern railroad for over twenty years, holding the position at the time of his death; and for about twelve years he was pension examining surgeon attached to the Buffalo board. He was a member of several medical societies, among which was the Medical Society of the State of New York, and he had been president of the Medical Society of the County of Erie.

Dr. Thompson was a representative physician in the region where he practised medicine and his services where often sought by his colleagues in the capacity of consultant. He was of commanding figure, of amiable disposition, skilful in his profession, an upright, honorable citizen and justly entitled to the grand old name of gentleman. His friends, neighbors, patients and colleagues were warmly attached to him and his memory will ever remain green in their hearts.

The funeral of Dr. Thompson was held at Angola, March 9, 1904, under the auspices of the masonic fraternity. The Rev. G. E. Henshaw delivered the funeral address, many persons from a distance being present. He is survived by a widow, one brother, one sister and a cousin, Norman R. Thompson, of Albany.

## Figures and Tables

**Figure f1:**